# Prevalence and factors associated with hepatitis b vaccination uptake and completion among communities targeted for mass vaccination in gulu: a cross-sectional study

**DOI:** 10.1186/s12889-024-18330-2

**Published:** 2024-03-20

**Authors:** Andrew Kimera, Lynn Atuyambe, Huzaifa Mutyaba, Claire Nantongo, Agnes Namagembe, Anna Maria Nalumansi, Andrew Basenero, Prisca Auma, Nelson Mukiza, Joan Mutyoba

**Affiliations:** 1 Makerere University College of Health Sciences, Kampala, Uganda; 2 RineCynth Advisory Limited, Kampala, Uganda

**Keywords:** Hepatitis B, Hepatitis B vaccination, Mass vaccination

## Abstract

**Background:**

Hepatitis B virus (HBV) is associated with several acute and long-term complications and vaccination is the cornerstone of prevention. A recent outbreak in Gulu, Uganda, one of the districts covered by a mass vaccination campaign, suggests low uptake of HBV vaccination. This study aims to determine the uptake and completion of HBV vaccination and associated factors among residents of Gulu, Uganda.

**Methods:**

A mixed methods cross-sectional study was conducted in Gulu, Northern Uganda, among 434 adult residents. A pretested questionnaire was used to collect data on socio-demographics, perceptions, and knowledge of HBV vaccination. Modified Poisson regression analysis was used in STATA 14 software to obtain prevalence ratios for the association between the independent and dependent variables. For qualitative data, 9 key informant interviews were conducted and thematic analysis was done using Quirkos software.

**Results:**

Out of the 434 respondents, 41.9% had received at least one dose of the hepatitis B vaccine, 32.5% had received at least 2 doses, and only 20% had completed all 3 doses, with an overall completion rate of 47.8% for participants who had been initiated on the vaccine. Gender, residence, risk perception of Hepatitis B infection, perceived safety of the vaccine, and awareness of mass vaccination were associated with uptake of Hepatitis B vaccination. Residence, knowledge, and perception of being at risk of acquiring Hepatitis B were associated with completion. Qualitative results revealed that the levels of uptake and completion could have been affected by access to vaccination sites; inadequate knowledge about the disease; myths about the vaccine and inadequate community engagement.

**Conclusion:**

Low Hepatitis B vaccine uptake and completion rates were observed in Gulu. To enhance vaccination coverage, future initiatives should prioritize awareness, education, and dispelling of vaccination myths. Additionally, increased government investment in training health workers can serve as a valuable strategy to improve information dissemination and awareness among the population.

## Introduction

The global prevalence of hepatitis B remains high at 3.9% with many people undiagnosed or not enrolled in treatment [1]. The prevalence and mortality registered in sub-Saharan Africa are even higher although the vaccine existed since 1982 [2]. The prevalence of HBV among the people of East Africa is estimated to be at 6.53% [3]. In Uganda, the prevalence of HBV infection stands at 4.1% among adults and 0.6% among children, with the highest rates observed in the mid-north region at 4.6% [4]. This emphasizes the need for mass vaccination [5].

Hepatitis B is a highly infectious and potentially lethal virus, causing acute liver failure, cirrhosis and fibrosis, Hepatocellular carcinoma, and ultimately death. The World Health Organization attributed 887,220 deaths in 2015 to HBV and its complications. In the year 2022, approximately 1250 Ugandans succumbed to Hepatitis B, and roughly 6% of the population continues to suffer from chronic infection [6]. Additionally, it is estimated that over 80% of individuals with chronic HBV infection are unaware of their status, missing out on clinical care, treatment, and interventions that are designed to reduce onward transmission [7]. Consequently, vaccination is the most efficient way of protecting populations against HBV, with a 3-dose regimen administered at intervals of 0, 1, and 6 months. Studies have shown that if the vaccine is completed, it is highly efficacious and confers lifelong protection on the individual [8]. In 2015, the government of Uganda started rolling out mass vaccination for highly endemic areas such as northern Uganda and also introduced the HBV vaccine as part of UNEPI to prevent infection in infants [9]. In 2018, it was reported that mass vaccination of the northern region was successfully completed [10].

Despite the completion of the mass vaccination effort, recent studies reveal a persistently higher prevalence of CHB in Northern Uganda compared to the national average (4.6% vs. 4.1%), as reported in the Uganda population-based HIV impact Assessment [11]. However, the previous assessments did not highlight completion rates or the factors influencing vaccine uptake. This study therefore seeks to explore uptake of HBV vaccines and associated factors in Gulu following mass vaccination.

## Methods

### Study design

A cross-sectional study using both quantitative and qualitative data collection methods was conducted between April and June 2022 to assess the uptake and completion rates of Hepatitis B vaccination and identify associated factors. Quantitative methods were used to measure the vaccination rates, while Key Informant Interviews were conducted in parallel to gain further insights into the factors that could have influenced these rates.

### Study setting

The research was carried out within the old boundaries of Gulu, which since July 1, 2020, now consists of Gulu city and Gulu district. Located in the Acholi sub-region in Northern Uganda, it has a population of 275,613 residents living in 70 parishes and 619 villages. Gulu has thirty-four health facilities, including four hospitals, two of which are government-owned, one HC IV, eleven HCIIIs, and sixteen HC IIs. The location was purposively selected because it still experiences outbreaks of hepatitis B after completing vaccination despite its high reported Hepatitis B vaccination coverage. Gulu City and Gulu District were chosen to represent urban and rural settings, respectively.

### Study population

The study considered individuals who were 18 years old and above, residing in both urban and rural areas of Gulu. The sampling frame included all members of household falling in this age group.

For qualitative data, the study considered individuals who were knowledgeable about the mass vaccination of 2015 or who were involved in the vaccination campaign. This included the district health team which was in-charge of the district campaign, health workers from Health Centre IVs where vaccines were distributed, Community Health Workers (CHWs) who participated in the mobilization, and local leaders from urban and rural Gulu.

### Eligibility

For quantitative data collection, we included all Gulu residents aged 18 and above who consented to the study and excluded those who were sick, mentally incapacitated, or unwilling to participate. Only key informants who held administrative or work position during the 2015 mass vaccination campaign were considered for the qualitative data collection.

### Quantitative sample size estimation

The sample size was determined using the Kish Leslie formula; n=$\left[ {{{{Z^2}PQ} \over {{\delta ^2}}}} \right]$, where Zα/2 = 1.96 (standard normal value at α = 5% level of significance), Where: n= desired sample size, z= standard normal deviation at 1.96 for a CL 95%, p= uptake of HBV vaccination in Soroti district, in Eastern Uganda reported to be 57.4% [12], d= permissible error at 5%, q= (1-p); resulting in a sample size of 375. Considering a 13% non-response rate (Average of non-response rate for academic research, 24% [13], and the non-response rate of the UDHIS 2016 household survey 2% [14]) N= n/ (1-0.13) = 375/ (1-0.13) = 434, a minimum sample size of 434 participants was considered for the study.

The principle of data saturation was used to determine the number of key informant interviews. Interviews were conducted until no new information emerged, resulting in a total of nine interviews.

### Sampling procedure

The 16 sub-counties in Gulu were divided into two categories: Gulu City, representing the urban population, and Gulu District, representing the rural population. A multi-stage sampling method was employed to select the study sample. Using the ballot method, a sub-county was randomly selected from Gulu City (Pece) and one from Gulu District (Paicho). Similarly, using the same method, two parishes were randomly chosen from each of the selected sub-counties. VanGaurd and Labourline parishes were selected from the urban area, while Omel and Pagik parishes were chosen from the rural areas. For the study, eight villages were selected randomly from each parish. The villages were chosen based on the population proportion, with the sample size being calculated from lists provided by LC1 chairpersons. The research team, working alongside the local chairperson, systematically selected households in each village. The first two eligible participants from each household who agreed to participate in the study were then interviewed.

Qualitative data was gathered through interviews with key informants. The informants were selected purposively to ensure representation of views from those responsible for the mass vaccination, those who mobilized the population, and other local leaders. The nine chosen key informants comprised of three administrators from the district health team, two health workers from HCIVs, three Community Health Workers (CHWs), and one LC1 chairperson.

### Data collection

Quantitative data was gathered using a pretested semi-structured questionnaire. The questionnaire was developed from scratch and then pretested to ensure the validity of the questions. The tool was then programmed into kobotoolbox.org for digital data collection and deployed on Kobo Collect® on mobile devices. The interviewer administered the tool to collect information on socio-demographic characteristics, knowledge, and perceptions of Hepatitis B vaccination.

Key Informant Interviews were conducted to collect qualitative data to reinforce the quantitative data. A Key Informant guide was designed guided by the Health Belief model theoretical framework for explaining and predicting health behavior [15]. These were recorded, transcribed and organized for analysis.

### Data analysis

Quantitative information collected was cleaned, organized and exported to Stata 14® for analysis. Knowledge was assessed using questions about HepB causes, transmission, complications and HepB vaccination. The questions asked were; (1)What causes Hepatitis B?; (2)List ways in which Hepatitis B can be transmitted; (3)List complications of Hepatitis B infection; and (4)List symptoms of Hepatitis B. Each collect response was score one point, giving a total possible score of 15. Participants were categorized as knowledgeable if they scored at least 70%.

The level of uptake was considered as the proportion of participants who had received at least one vaccine dose. Completion was the proportion who had received all the recommended 3 doses among those who had received at least a dose of the HepB vaccine. Bivariate analysis was performed and factors that achieved a p value ≤ 0.2 were considered for model building at the multivariate level using modified Poisson regression. Collinearity was assessed before coming up with the model. Factors that achieved a p-value less than or equal to 0.05 were considered to be significantly associated with Hepatitis B vaccine uptake and completion.

The qualitative data was transcribed and then analyzed using Quirkos® software. Inductive thematic analysis was used to identify themes on factors associated with the uptake and completion of the HepB vaccine. First, the transcripts were thoroughly read to become familiar with the data, and then coding was done. The codes were used to generate themes, which were reviewed and renamed as needed.

## Results

Quantitative data from 434 respondents was analyzed and the median age of participants was 33 (IQR: 25–50) years. More than half of them were females; 246(56.7%), and the majority lived in urban areas; 279 (64.3%). Only 119(27.4%) of the individuals were considered knowledgeable about Hepatitis B(HepB). A little over half of the individuals believed they were at risk of getting Hepatitis B; 233(53%), and most knew about the mass vaccination campaign; 326 (75.1%). A large portion of the study participants believed the vaccine was effective 287(66.1%), and 291(67.1%) individuals thought it was safe.

### Uptake and completion of Hepatitis B vaccination in Gulu

Out of the 434 participants, 182 had received at least one dose of HepB vaccination 41.94% (95% CI: 37.4–46.7). Uptake was higher in the urban Gulu, 48.0% (95% CI: 42.2–53.9) compared to the rural Gulu 30.9% (95% CI: 24.2–38.7).

Out of the 182 participants who had started on HepB vaccination, 87 completed the recommended 3 doses yielding a completion rate after the first dose of 47.8% (95% CI: 40.6–55.1). Completion rates were higher in urban Gulu, 53.7% (95% CI: 45.2–62.1) compared to rural Gulu, 31.1% (95% CI: 19.6–45.8).

### Reasons given for not receiving Hepatitis B vaccination or not completing vaccination among participants from Gulu

**Fig. 1:**
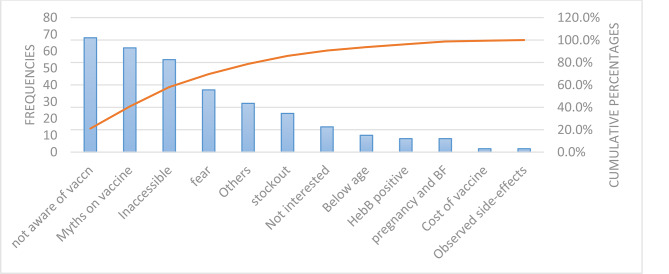
Pareto chart showing reasons given for not being vaccinated

**Fig. 2:**
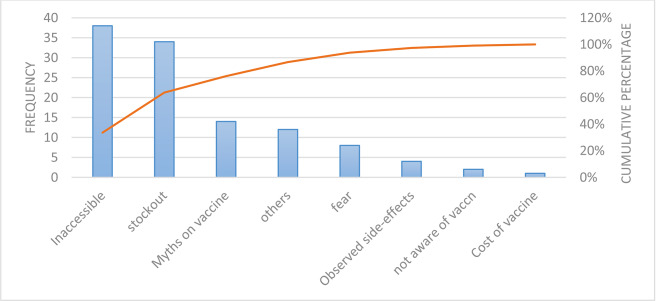
Pareto chart showing reasons given for receiving less than 3 doses

The major reasons given by 80% of the participants for non-uptake of the Hepatitis B vaccine included a lack of awareness about the vaccination campaign, myths about the vaccine, inaccessibility of the vaccine, and fear (Fig. 1). Regarding the non-completion of the full three doses of the Hepatitis B vaccine, 80% of participants attributed it to challenges such as difficulties accessing vaccination sites, stockouts, and myths about the vaccine (Fig. 2).

### Factors associated with uptake of hepatitis B vaccination in Gulu

Modified Poisson regression in the bivariable analysis revealed associations between Hepatitis B vaccine uptake and various factors including; sex, residence, education level, employment status, knowledge about Hepatitis B, perceived risk of Hepatitis B, perceived vaccine safety and effectiveness, as well as awareness of the mass vaccination campaign (Table 1). At multivariable analysis, gender, residence, perceived risk of Hepatitis B, perceived safety of vaccine, knowledge of Hepatitis B, and awareness of mass vaccination were significantly associated with uptake of the hepatitis B vaccine (Table 1).

Females were 35% more likely to get at least one dose of vaccination than males, Adj.PR: 1.31 (1.08–1.6). Urban residents had a 47% higher likelihood of taking up the vaccine compared to rural residents, Adj.PR: 1.47 (1.16–1.86). Respondents who were knowledgeable about Hepatitis B were 25% more likely to take up the vaccine than those who were not knowledgeable about Hepatitis B, Adj.PR: 1.25 (1.04–1.50). Participants who reported to have been aware of the mass vaccination campaign were 2.38 times more likely to have received at least one dose of the vaccine compared to those who were not aware Adj. PR: 2.38 (1.53–3.72). Individuals who perceived themselves to be at risk of hepatitis B infection were 27% less likely to have taken up vaccination compared to those who did not perceive themselves to be at risk or those who did not know if they were at risk, Adj.PR: 0.73 (0.61–0.88). Those who believed that the vaccine was safe were almost 3 times more likely to take up the vaccine than those who did not, Adj. PR: 2.71 (1.2–6.08).

**Table 1:** Bivariate and multivariate analysis of factors associated with uptake of hepatitis B vaccination

	Ever vaccinated		CPR (95% CI)	p-value	APR (95% CI)	p-value
	NO Freq (%)	YES Freq (%)				
**Sex**						
Male	121 (64.4)	67 (35.6)	1		1	
Female	131 (53.3)	115 (46.7)	1.31 (1.04–1.66)	**0.023**	1.31 (1.08–1.6)	0.003**
**Tribe**						
Acholi	237 (59.1)	164 (40.9)	1			
Others	15 (45.5)	18 (54.5)	1.33 (0.96–1.86)	0.09		
**Residence**						
Rural	107 (69)	48 (31)	1		1	
Urban	145 (52)	134 (48)	1.55 (1.19–2.02)	**0.001**	1.47 (1.16–1.86)	0.001**
**Education level**						
None	22 (64.7)	12 (35.3)	1			
Primary	134 (68)	63 (32)	0.91 (0.55–1.49)	0.699		
Secondary	70 (50.4)	69 (49.6)	1.41 (0.87–2.29)	0.169		
Tertiary	26 (40.6)	38 (59.4)	1.68 (1.02–2.77)	**0.041**		
**Employment**						
Unemployment	26 (53.1)	23 (46.9)	1			
Peasant farmer	104 (69.8)	45 (30.2)	0.64 (0.44–0.95)	**0.025**		
Self-employed	98 (51.3)	93 (48.7)	1.04 (0.74–1.45)	0.829		
Formal employment	24 (53.3)	21 (46.7)	0.99 (0.65–1.53)	0.979		
**Estimated monthly income (Ugx)**						
< 0.1 m	108(66.7)	54(33.3)	1			
0.1-<0.3 m	91(54.8)	75(45.2)	1.36(1.03–1.78)	**0.03**		
At least 0.3 m	53(50)	53(50)	1.5(1.12-2.00)	**0.006**		
**Knowledge of Hepatitis B**						
Not knowledgeable	204 (64.8)	111 (35.2)	1			
Knowledgeable	48 (40.3)	71 (59.7)	1.69 (1.37–2.09)	**< 0.001**		
**Perceived risk**						
No/don’t know	105(52.2)	96(47.8)	1		1	
Yes	147(63.1)	86(36.9)	0.77(0.62–0.96)	**0.023**	0.73 (0.61–0.88)	0.001**
**Perceived Hepatitis B vaccine effectiveness**						
No/don’t know	124(84.4)	23(15.6)	1			
Yes	128(44.6)	159(55.4)	3.54(2.40–5.23)	**< 0.001**		
**Perceived Safety of Vaccine**						
Unsafe/ don’t know	127(88.8)	16(11.2)	1		1	
Safe	125(43)	166(57)	5.1(3.18–8.18)	**< 0.001**	4.24 (2.66–6.77)	< 0.001***
**Knowledge**						
Not knowledgeable			1		1	
Knowledgeable			1.69 (1.37–2.09)	**< 0.001**	1.25 (1.04–1.50)	0.017*
**Awareness of mass vaccination**						
No	94 (87)	14 (13)	1		1	
Yes	158 (48.5)	168 (51.5)	3.98 (2.41–6.56)	**< 0.001**	2.53 (1.59–4.03)	< 0.001***

CPR = crude prevalence level; APR = adjusted prevalence level; *<0.5 **0.01 − 0.001 ***<0.001

### Factors associated with completion of hepatitis B vaccination in Gulu

Bivariate analysis using modified Poisson regression revealed associations between completion of Hepatitis B vaccination and factors such as residence, employment status, income, and perceived risk of Hepatitis B (Table 2). At multivariate analysis, urban residence, knowledge of Hepatitis B, and a perception of being at risk of acquiring Hepatitis B were statistically significantly associated with completion of the hepatitis B vaccine after taking the first dose (Table 2).

Urban residents had a 52% higher likelihood of completing the vaccine compared to rural residents, Adj.PR: 1.52 (1.01–2.31). Respondents who were knowledgeable about Hepatitis B were 44% more likely to complete vaccination than those who were not knowledgeable about Hepatitis B, Adj.PR: 1.44 (1.12–1.86). Individuals who perceived themselves to be at risk of hepatitis B infection were 64% less likely to have completed the 3 doses compared to those who did not perceive themselves at risk or those who did not know if they were at risk, Adj.PR: 0.36 (0.24–0.54). It is however important to note that sex, perceived safety of the vaccine, and awareness of the mass vaccination campaign were not independently associated with completion of vaccination.

**Table 2:** Bivariate and Multivariate analysis of factors associated with completion of hepatitis B vaccination

	Completion of vaccination					
	NO Freq (%)	YES Freq (%)	CPR (95% CI)	p-value	APR (95% CI)	p-value
**Residence**						
Rural	33(68.8)	15(31.3)	1		1	
Urban	62(46.3)	72(53.7)	1.72(1.10–2.69)	**0.018**	1.52 (1.01–2.31)	0.046*
**Education level**						
None	33(68.8)	15(31.3)	1			
Primary	62(46.3)	72(53.7)	0.88(0.42–1.85)	0.729		
Secondary	33(68.8)	15(31.3)	1.18(0.58–2.41)	0.645		
Tertiary	62(46.3)	72(53.7)	1.58(0.78–3.21)	0.207		
**Employment**						
Unemployment	13(56.5)	10(43.5)	1			
Peasant farmer	32(71.1)	13(28.9)	0.66(0.34–1.28)	0.222		
Self-employed	46(49.5)	47(50.5)	1.16(0.70–1.93)	0.562		
Formal employment	4(19)	17(81)	1.86(1.12–3.11)	**0.017**		
**Estimated monthly income(ugx)**						
< 0.1 m	108(66.7)	54(33.3)	1			
0.1-<0.3 m	91(54.8)	75(45.2)	1.19(0.77–1.83)	0.435		
At least 0.3 m	53(50)	53(50)	1.73(1.16–2.59)	**0.008**		
**Awareness of mass vaccination**						
No	7(50)	7(50)	1		1	
Yes	88(52.4)	80(47.6)	0.95(0.55–1.65)	0.862	1.17 (0.7–1.96)	0.556
**Knowledge of Hepatitis B**						
Not knowledgeable	67(60.4)	44(39.6)	1		1	
Knowledgeable	28(39.4)	43(60.6)	1.19(0.77–1.83)	0.435	1.44 (1.12–1.86)	0.005*
**Vaccine safety**						
Unsafe/ Don’t know	127(88.8)	16(11.2)	1			
Safe	125(43)	166(57)	2(0.84–4.75)	0.116		
**Perceived risk**						
No/don’t know	105(52.2)	96(47.8)	1		1	
Yes	147(63.1)	86(36.9)	0.36(0.24–0.53)	**< 0.001**	0.36 (0.24–0.54)	< 0.001*

CPR = crude prevalence level; APR = adjusted prevalence level; *<0.5 **0.01 − 0.001 ***<0.001

### Qualitative results

The following themes emerged from qualitative analysis.

### Limited access to vaccination sites

More than half of the key informants believed that the vaccination sites were not very convenient, especially for the rural population. They reported that the campaign was run from HCIVs and above and that some communities were far from vaccination sites.

*“We are very far from the big hospital so they delayed to come to our village here.” [Local leader]*.

### Awareness of Hepatitis B

All the administrators and one VHT felt that the people did not fully understand Hepatitis B or appreciate the benefits of being fully vaccinated for hepatitis B.

*“The disease is considered new and has only been known after it has killed some people.” [Administrator 1]*.

*“Few who picked it up got only the first dose and didn’t know the importance of coming back for the second and third doses.” [Administrator 2]*.

### Myths on vaccination

All the key informants believed myths about vaccines as well as the fear of side effects affected uptake and completion during the mass vaccination campaign.

*“People were so concerned about vaccine safety, the side effects, how they would react so that alone I believe it was a factor why some people never came for the vaccine.” [Administrator]*.

*“The men fear thinking that when you test negative for hepatitis B and then get vaccinated, the next time you will test positive.” [Health worker]*.

The majority of the key informants believed many myths about the vaccine stemmed from mistrust of the government thus affecting uptake.

*“From the time of insurgency, people don’t trust the government. Anything that comes, people think the government has come to destroy, regardless of it being good.” [Health worker 1]*.

### Inadequate community engagement

Many key informants cited inadequate community engagement during the campaign. They believed there was a proportion of the population who did not know about the campaign and therefore missed out on the vaccination.

*“We didn’t have enough information. We delayed to get information on vaccination which affected uptake*.*” [Local leader]*.

## Discussion

Hepatitis B vaccination uptake was 41.9%, reflecting the low HBV vaccine uptake among adults in Uganda, potentially contributing to the recent HBV outbreak in Gulu, despite mass vaccination coverage. However, this study’s uptake rate surpasses figures from previous African studies, such as 14.2% in Nigeria [16] and 4% among Ethiopian health workers [17]. Conversely, a Ghanaian study reported a higher uptake of 53.4% [18] than observed in this study. This is likely due to its hospital-based nature and regional differences in customs, belief systems, and vaccination interventions. Enhancing future vaccine uptake necessitates community sensitization, dispelling HBV vaccination myths, and improving accessibility to vaccination services.

Completion of Hepatitis B vaccination among adults in Gulu was low, with less than half (47%) of those who started the vaccine completing all three doses. While this rate falls below the 52% completion rate observed in hard-to-reach populations in London [19] and the 57.8% completion among health workers in Wakiso district [20], it surpasses the 35% completion in a UK cohort with a 36-month follow-up [21], where data entry errors may have played a role. Moreover, it exceeds the 30.5% completion rate observed among public safety workers in Nigeria [22], who share similarities with the Gulu community’s high-risk profile for Hepatitis B. Notably, the main barriers to completion were inaccessibility of vaccination points and stockouts, highlighting the importance of ensuring accessibility to testing points and a ready vaccine supply in future vaccination campaigns.

Females were more likely to get at least one vaccine, which was potentially due to the better health-seeking behavior of women compared to men in this population, especially considering the vaccines were mostly provided at health facilities rather than community outreaches. Urban residents were more likely to take up and complete vaccination compared to the rural participants. This could have been due to more vaccination sites having been stationed in the urban areas than the rural areas as reported by the key informants. Distance to vaccination sites was mentioned as being a major barrier to vaccination, especially among rural communities. It is therefore not surprising that one of the major reasons the study participants gave for either not getting a vaccine or not completing the recommended doses was inaccessibility of the vaccination sites. The HBV service providers should therefore bring their services closer to communities to ensure easy access to the vaccination sites.

The one reason the majority of participants who did not get any vaccine mentioned was not being aware of the campaign. Participants who were reported to have been aware of the mass vaccination campaign were twice as likely to have received at least one dose. The limited sensitization probably failed to address knowledge gaps on the virus and vaccination. This is important in targeting people’s perceptions of the risk to the disease and safety of the vaccines as well as addressing myths circulating in the community. Belief in the safety of the vaccine was associated with 2.8 times higher likelihood of getting at least one dose of the vaccine. Of the people who completed it, none felt the vaccine was unsafe.

In this study, knowledge of Hepatitis B was a predictor of uptake and completion. These findings are similar to those by Chingle et al. [23] and Meriki et al. [24] who suggested that knowledge of disease and vaccination, could be a factor associated with HBV vaccine uptake. These findings contradict those from studies done in the USA, Cameroon, Ghana, and Nigeria that showed no relationship between knowledge and uptake [25]. A study conducted in Kenya among health workers showed that while 78.6% had good knowledge of Hepatitis B only 59.6% had been vaccinated and of these, only 32.0% had completed the recommended three-dose vaccination [26]. This means that knowledge on its own may not be adequate to promote uptake and completion and may need to be complemented with other strategies.

Our study also found that participants who felt they were not at risk of contracting hepatitis B were more likely to have received at least one dose of the vaccine or completed vaccination compared to those who felt they were at risk. The other reason could be that they were answering in regards to the current context being that the vaccination was done more than 5 years ago. They therefore felt that since they had received at least one dose of the vaccine, they were not at risk of hepatitis B infection.

Regarding health system factors that contributed to the low uptake and completion were stock out and lack of continuity. Key informants mentioned stock out of vaccines and test kits in some facilities to have posed a big challenge. It is not surprising that one of the major reasons survey participants gave for not completing their doses was stock out of vaccines. After the vaccination campaign, the vaccines were reportedly not availed for routine vaccination. This probably means that when late adopters wanted to take up vaccination or complete their doses, they could not access the free vaccines from any facilities in the area. This calls for proper planning on the part of the service providers to ensure the continuity of the service by minimizing stockouts.

### Study strengths and limitations

This study, to our knowledge, is the first in Uganda to investigate Hepatitis B vaccination uptake and completion following a mass campaign, shedding light on influencing factors, and giving a benchmark for further research and other recommendations to MOH for nationwide implementation. Nevertheless, it has some limitations. Factors such as knowledge and perception may have changed over time, potentially differing from the pre-vaccination campaign period. Reported information on vaccine uptake and completion couldn’t be independently verified as the many participants had lost their vaccination cards. Additionally, recall bias could have been introduced because of the time period between the mass vaccination and the study. Moreover, selection bias at the household level could have impacted outcomes, particularly in factors like knowledge and perceived seriousness.

### Conclusion and recommendations

Hepatitis B uptake and completion rates were low in Gulu, with only 41.9% receiving at least one dose, and of those, only 47.8% completing the recommended 3 doses. Gender, residence, perceived Hepatitis B risk, vaccine safety perception, knowledge and awareness of the mass vaccination campaign independently influenced vaccination uptake, while residence, knowledge, and perceived Hepatitis B risk independently affected completion. Qualitative results revealed reasons for vaccine non-uptake and non-completion to be; limited access to vaccination sites, inadequate knowledge about the disease and low appreciation for benefits of vaccination, concerns about vaccine safety, limited vaccine awareness, health worker knowledge gaps, and stockouts. Streamlining mass vaccination campaigns to ensure all regions are covered, sensitizing healthcare workers and communities, and ensuring adequate mobilization can contribute to Uganda’s efforts to reduce the national prevalence of hepatitis B virus infection and achieve the 2030 global goals for HBV reduction.

## Data Availability

Data used in this study is available upon request from the corresponding author.

## Abbreviations

CDC: Centers for Disease Control
CHB: Chronic Hepatitis B
HBsAg: Hepatitis B Surface Antigen
HBeAg: Hepatitis B Envelope Antigen
HepB: Hepatitis B
HBV: Hepatitis B Virus
HIV: Human Immunodeficiency Virus
H/C: Health Centre
MOH: Ministry of Health Uganda
OPD: Outpatients Department
STD: Sexually transmitted Disease
TB: Tuberculosis
UK: United Kingdom
UNEPI: Uganda National Expanded Program on Immunization
WHO: World Health Organization
